# Osteogenesis imperfecta Type IV: a newly identified variant at position c.560 (G > T; p.Gly187Val) in the COL1A2 gene

**DOI:** 10.11604/pamj.2017.27.198.12295

**Published:** 2017-07-14

**Authors:** Akin Usta, Dilay Karademir, Eylem Sen, Selcuk Yazici, Ertan Adali, Erkan Erdem, Meric Karacan

**Affiliations:** 1 Department of Obstetrics and Gynecology, School of Medicine, Balikesir University, Balikesir,Turkey; 2 Department of Pediatrics, Balikesir Ataturk State Hospital, Balikesir,Turkey; 3 Department of Pediatrics, School of Medicine, Balikesir University, Balikesir,Turkey; 4 Department of Reproductive Endocrinology and Infertility, Ota-Jinemed Hospital, Istanbul, Turkey

**Keywords:** Osteogenesis ιmprefecta, skeletal dysplasia, malecular analysis, COL1A2 gene

## Abstract

Osteogenesis imperfecta is a clinically heterogenous disease caused by defective collagen syntesis associated with a mutation in the COL1A1 or COL1A2 genes. In this report, we present a case of osteogenesis imperfecta (OI) type IV, seen in a female fetus with incurved femurs at 18 weeks of gestation. Molecular analysis of the newborn revealed a novel mutation at position c.560 (c.560 G > T) of the exon 12 in the COL1A2 gene; which lead to the glycine modification with valine (p.Gly187Val) at codon 187. The pregnancy follow-up was uneventful. After delivery, the newborn underwent biphosponat therapy and no fracture was detected until 1 year old.

## Introduction

Osteogenesis imperfecta (OI) is a hereditary disease characterised by osteopenia, bone deformity and fracture, hyperlaxity of ligaments and skin, short stature, hearing loss, blue sclera, and dentinogenesis imperfecta [1, 2]. It is very rare with a prevalence of about 6-7 per 100,000 births [3]. Genetically, many causative mutations in 19 different genes have been described in the variant database [4]. Although autosomal recessive inheritance can be seen in some cases with OI, in the majority of cases the transition is autosomal dominant with mutation in COL1A1 or COL1A2 genes, encoding the alpha 1 and 2 chains of collagen type I [5]. Sillence et al. first identified genetic heterogenity in patients with OI in 1979 and classified into 4 syndromic subgroups [6]. Currently, 5 types of OI are accepted for clinical description. The severity of the disease varies from nearly asymptomatic individuals to perinatal lethality in individuals with multiple fractures [2]. OI type IV is a mild form that can rarely be detected by showing long bone deformities with ultrasonography especially in the 2^nd^ or 3^rd^trimester. Clinical findings of OI type IV include mild/moderate extremity bending with or without fractures, short stature, dentinogenesis imperfecta and hearing loss [7]. Herein, we present a new case of OI type IV with a newly detected mutation in the COL1A2 gene presented with incurved femurs of a female fetus in second trimester of gestation.

## Patient and observation

**Figure 1:**
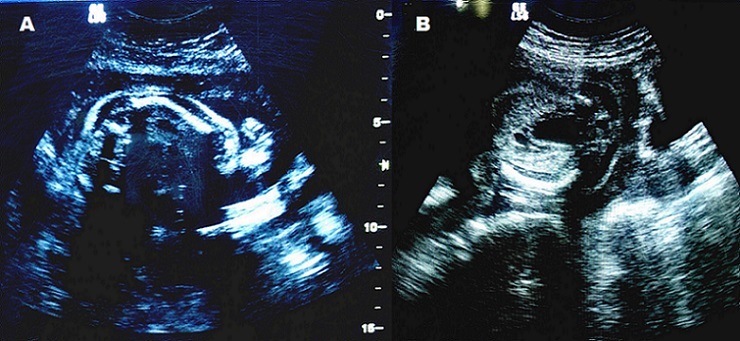
Prenatal ultrasonography at 18 weeks of gestation: (A) curved right femur; (B) curved left femur

**Figure 2:**
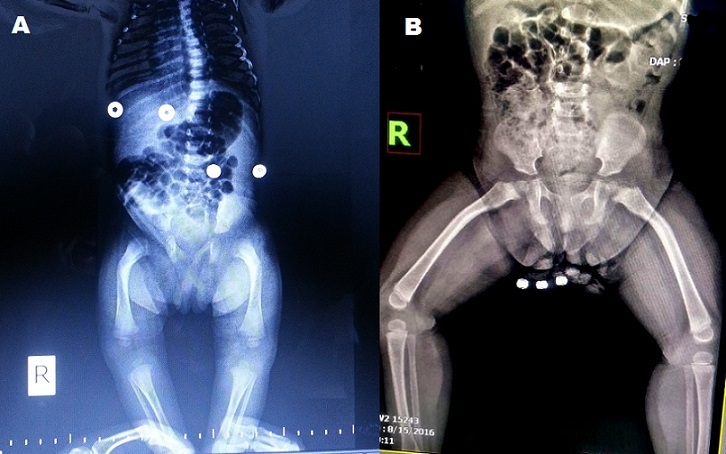
Postnatal radiography shows mildly curved femurs and osteopenia: (A) First day after birth, (B) One year later after birth

A 32-year-old woman (gravida 1, parity 0) was admitted to the hospital for prenatal follow-up in the first trimester. There was no significant medical history. The woman's body weight was 58 kg, height was 154 cm. Her husband had no OI and height was 159 cm. First trimester screening was done at 12w+3d of pregnancy. Fetal nuchal translucency thickness was 2.1 mm at that time. Pregnancy-associated plasma protein-A and human chorionic gonadotropin (HCG) were measured in the mother's blood sampling and no abnormal results were detected (1.2 mom and 0.9 mom, respectively). Second trimester ultrasound showed incurved femurs of the fetus at 18 weeks of gestation (Figure 1). Parents were given genetic counseling and amniocentesis was recommended. Parents refused amniocentesis and decided to carry their pregnancy until term. Prenatal ultrasonography in the third trimester showed that the biparietal diameter and abdominal circumference were equivalent to 38 weeks. However, right and left femurs were measured 6.2 and 6.3 cm, respectively (< 2 SD). A cesarean section was performed at 39th gestational week for breech presentation, and a 2630 gram female baby with a body length of 48 cm was delivered. On postnatal radiography, mild osteopenia and the curvature of the femurs were detected (Figure 2A). Molecular analysis of the newborn showed that a G to T change at position c.560 (c.560 G > T) of the exon 12 in the COL1A2 gene; which lead to the glycine modification with valine (p.Gly187Val) at codon 187. Orally informed consent was taken from patient.

## Discussion

In this case report, we identified OI in a female fetus, presented with bilaterally incurved femurs at the 18 weeks gestations. Molecular analysis of the newborn revealed a novel mutation at position c.560 (c.560 G > T) of the exon 12 in the COL1A2 gene; which led to the change of glycine with valine (p.Gly187Val) in codon 187. The pregnancy follow-up was uneventful and the skeletal system of the newborn was not broken until 1 year old (Figure 2B). To date, 589 different pathogenetic variants have been reported in COL1A2 gene in OI patients [4]. Previously, mutation at the position c.560, G>A was described as a pathogenic substitution in two cases (one case was classified as type I OI, the other as type IV OI) with osteogenesis imperfecta in the variant database; however, G>T change has not been reported yet in the variant database. OI is a hereditary bone disease caused by defective collagen synthesis. Collagen is the most abundant protein responsible for the structural integrity of multicellular organisms and found in the extracellular matrix of connective tissue. Each of the collagen types has different functions and properties. They also exhibit a characteristic tissue distribution. Type 1 collagen is the major collagen type, existing in most tissues such as bone, skin and tendons. It constitutes about 95% of the entire collagen in the bone and is responsible for the strength of bone tissue, especially against resistance, slippage or compaction [8]. Type 1 collagen is formed by three polypeptide chains (alpha-chains) that form a triple helix. For a triple helix configuration (Gly-X-Y triple), glycine is an absolute requirement in every third position because glycine has the property of being the smallest amino acid that can cover the confined space in the center of the triple helix. X can be any amino acid, but is usually a proline, while Y is usually a hydroxyproline [6]. People with OI type IV usually show structural abnormalities in the triple helix of type 1 collagen due to point mutations, insertions, deletions, or splice mutations in the COL1A2 gene. Previous studies have reported that helical glycine mutations in COL1A1/COL1A2 genes were associated with heavier bone fragility and damage than other type of mutations such as haploinsufficiency in COL1A1/COL1A2 genes [8, 9]. However, some cases reported were related to helical glycine mutation in COL1A1/COL1A2 genes with favorable outcomes [10]. In the present case, the newborn had helical glycine mutation in COL1A2 gene with a favorable outcome. Moreover, approximately one year the baby underwent biphosponat therapy after delivery and no fracture was detected.

In patients with OI, skeletal dysplasia is most often seen in long bones, a symptom caused by defective healing process after a fracture, or distortion of the bone without fracture. Other bone deformities commonly seen in OI patients are scoliosis, vertebral deformities, and compression fractures that occur with similar mechanisms [11]. Early detection of bone deformities during pregnancy is believed to be associated with severity of the disease. Only severe and lethal forms of OI such as type 2 and type 3 can be detected by ultrasound before 20 weeks gestation [12]. Previous studies showed that only 58% of patients with OI present with bone deformities [13]. The mild forms of OI such as type 1 and type 4 are detected either in late gestation when upon diagnosis of skeletal deformities or in the neonatal period [14]. In the present case, femoral bowing was detected at 18 weeks of gestation. This suggests that early detection of skeletal deformity may not be related to the progression of the disease. Early prenatal diagnosis of OI is possible through prenatal genetic testing by CVS and amniocentesis either following the detection of bone deformities on ultrasound or upon the history of familial OI without bone deformities in mild cases. Parental molecular analysis or the detection of mutated genes and mutation type before the delivery can be offered in applicable cases. It should also be emphasized that a wide range of abnormalities pertinent to different genotypes may be detected.

## Conclusion

A novel mutation of the exon 12 in the COL1A2 gene was detected in a newborn with OI that was diagnosed with bilateral incurved femurs at 18 weeks of gestation. According to our knowledge, OI type IV is a mild form that can rarely be detected by showing long bone deformities with ultrasonography during the prenatal examination. Presented case is an example that the early prenatal diagnosis of skeletal deformity may not indicate the progression of the disease in patients with OI.

## Competing interests

The authors declare no competing interests.
